# Choroid plexus tumours

**DOI:** 10.1038/sj.bjc.6600609

**Published:** 2002-11-04

**Authors:** J E A Wolff, M Sajedi, R Brant, M J Coppes, R M Egeler

**Affiliations:** Department of Pediatric Oncology, Alberta Children's Hospital, 1820 Richmond Road SW, Calgary, AB, Canada T2T 5C7; Klinik St. Hedwig, Hämato/Onkologische Abteilung, Steinmetzstr. 1 – 3, 93049 Regensburg, Germany; Department of Epidemiology, University of Calgary, 2500 University Drive NW, Calgary, AB, Canada T2N 1N4

**Keywords:** brain tumour, meta-analysis, prognostic factors, treatment, survival, irradiation

## Abstract

Choroid plexus tumours are rare epithelial brain tumours and limited information is available regarding their biology and the best treatment. A meta-analysis was done to determine prognostic factors and the influence of various treatment modalities. A thorough review of the medical literature (1966–1998) revealed 566 well-documented choroid plexus tumours. These were entered into a database, which was analysed to determine prognostic factors and treatment modalities. Most patients with a supratentorial tumour were children, while the most common sites in adults were the fourth ventricle and the cerebellar pontine angle. Cerebellar pontine angle tumours were more frequently benign. Histology was the most important prognostic factor, as one, five, and 10-year projected survival rates were 90, 81, and 77% in choroid plexus-papilloma (*n*=353) compared to only 71, 41, and 35% in choroid plexus-carcinoma respectively (*P*<0.0005). Surgery was prognostically relevant for both choroid plexus-papilloma (*P*=0.0005) and choroid plexus-carcinoma (*P*=0.0001). Radiotherapy was associated with significantly better survival in choroid plexus-carcinomas. Eight of 22 documented choroid plexus-carcinomas responded to chemotherapy. Relapse after primary treatment was a poor prognostic factor in choroid plexus-carcinoma patients but not in choroid plexus-papilloma patients. Treatment of choroid plexus tumours should start with radical surgical resection. This should be followed by adjuvant treatment in case of choroid plexus-carcinoma, and a ‘wait and see’ approach in choroid plexus-papilloma.

*British Journal of Cancer* (2002) **87**, 1086–1091. doi:10.1038/sj.bjc.6600609 www.bjcancer.com

© 2002 Cancer Research UK

## 

Choroid plexus tumours (CPTs) are relatively rare primary brain tumours arising from epithelial differentiated tissue. With an annual incidence of 0.3 cases per million (Janisch and Staneczek, 1988, 1989), these tumours account for only 0.4–0.8% of all brain tumours (Zülch, 1957). The most commonly reported locations are the ventricles. Nevertheless, extraventricular sites have been described (Kimura *et al*, 1992; Steven *et al*, 1996; Mottl *et al*, 1997; Nakano *et al*, 1997), too. Tumours are classified as choroid plexus-carcinoma (CPC, WHO grade III) (Kleihues and Canvanell, 2000), when they show nuclear pleomorphism, high nucleus to cytoplasm ratios, blurring of the papillary pattern, and necrosis (Paulus and Janisch, 1990). In addition, these tumours frequently express carcinoembryonic antigen (CEA) (Paulus and Janisch, 1990), and CD44 (Varga *et al*, 1996) which can also be detected as a marker in peripheral blood (Hashizume *et al*, 1995). Choroid plexus-papilloma (CPP, WHO grade I), the more differentiated tumours, expresses more frequently the markers pre-albumin (Matsushima *et al*, 1988) and the S100 protein. The average labelling indices with MIB1, a proliferation marker, were 14% in CPC and 3.7% in CPP (Vajtai *et al*, 1996). However, this histological classification is not clear cut. The possibility of a development from papilloma to carcinoma has been documented (Diengdoh and Shaw, 1993), and metastases occur not only in patients with CPC (Bennedback and Therkildsen, 1990; Kang *et al*, 1997) but also in patients with CPP (Leys *et al*, 1986; Domingues *et al*, 1991; Enomoto *et al*, 1991).

The current treatment of choroid plexus tumours is based on little evidence. None of the national paediatric oncology groups cover a population large enough to perform a randomised trial. In general it is believed that surgical resection is an important element of treatment. Postoperatively, infants with choroid plexus carcinoma are frequently treated with adjuvant chemotherapy and adult patients are treated with irradiation therapy. Patients with choroid plexus papilloma are mostly treated similar to those with low grade glioma. We performed a systematic literature review to assess prognostic factors and treatment efficacy in choroid plexus tumours as a basis for stimulating an international consensus to form the basis of an international trial working group mainly within the International Society for Paediatric Oncology (SIOP).

## METHODS

The first step for this analysis was a literature search in the Medline database, including all publications up to January 1998. Search words used were ‘Choroid plexus’ or ‘CPT’ or ‘CPP’ limited to ‘human’. This yielded 4120 titles of which 1520 appeared promising to contain the information listed below. Reading the abstracts reduced this number to 551. However, reading those publications increased the number again, because the referee list of some of them contained publications, which had not been found in Medline. Some of those were chapters in textbooks (Rubinstein and Brucher, 1981; Kleihues *et al*, 1997). This yielded binally a total of 572 publications.

The second step was to create a patient database from cases mentioned in the literature. Documented fields included first author, publication year, symptoms and signs, duration of symptoms until diagnosis, age at diagnosis, race, gender, histology, size of tumour, location, presence of metastases, type of surgery, type of radiotherapy, radiotherapy response, type of chemotherapy, chemo-response, tumour recurrence, observation time, and outcome. When tumours were diagnosed at autopsy or when patients died during the first surgery, they were encoded as observation time=0 and outcome=death. Patients published without any observation time and without outcome description were encoded as observation time=0, outcome=survival. Not all of these fields collected sufficient information for final analysis. Publications were excluded from the analysis when extractable information filled less than two fields of the database. In case of replicated publications from the same group, only the most recent publication was used for data-entry.

The database was first analysed using the technique of the stratified subgroup analyses with ‘SPSS®’ (SPSS Inc, San Francisco, CA, USA). First the qualitative parameters such as tumour location, histology, and gender were used to divide the database in various groups. χ^2^ or Fishers exact tests and ANOVA were used to assess the homogeneity for other parameters within these groups and subgroups. Survival was analysed using Kaplan–Meier estimates and compared with log rank tests. Cox regression analysis was then used to repeat analyses for the same questions.

## RESULTS

After applying the exclusion criteria, 217 publications were left with sufficient information. The number of patients reported within each study was between one and 54 (Palazzi *et al*, 1989). This resulted in a database of 566 patients.

**Figure 1:**
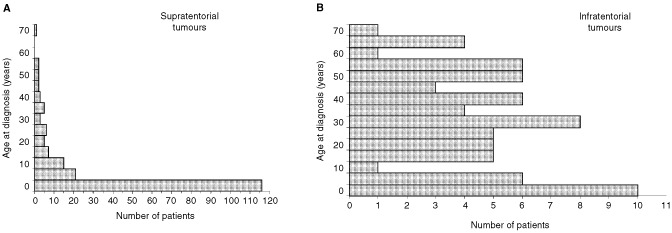
Age distribution of choroid plexus tumours: Supratentorial tumours occur most frequently in infants (A) *n*=188, while the age distribution of infratentorial tumours is spread over all ages (B), *n*=71; *P*<0.005, Mann–Whitney *U*-test). This analysis included only patients with documented primary intracerebral locations.

Gender, age at diagnosis, location, histology and metastases could be correlated to each other regardless if treatment or observation time data were sufficient. Surprisingly, none of these parameters was distributed homogeneously among groups defined by the others. In the total group, the male : female ratio was 1.2 : 1. Relating gender to location showed relatively more female patients with tumours located in the cerebellopontine angle (CPA, 29 observed, 20 expected). Age at diagnosis covered a large range from 0 years (foetus) to 72 years, but most of the patients were children, resulting in a median age at diagnosis of 3.5 years in the whole database. The most striking inhomogeneity was found when relating age to location (Figure 1

). The age distribution shifted from younger to older patients the more caudal the tumour was located. This resulted in median ages of 1.5 years, 1.5 years, 22.5 years, and 35.5 years in the groups of tumours located in the lateral ventricle, third ventricle, fourth ventricle and CPA, respectively (*P*<0.005 ANOVA). In the total group, the number of CPP was higher than CPC (353 *vs* 207). When relating malignancy to location, the relative frequency of CPC was higher in lateral ventricles and CPP was more frequent in CPA-tumours (*P*<0.005). Overall 12% of patients presented with metastatic disease. This frequency was higher for those with CPC histology (*P*<0.0005) and with supratentorial location (*P*=0.04). However, numbers were too small for subgroup analyses of this variable. The metastases occurred predominantly along the cerebrospinal fluid pathways (Allen *et al*, 1992), other sites included abdomen (Geerts *et al*, 1996), bone (Valladares *et al*, 1980), and lung (Sheridan and Besser, 1994). Abdominal seeding has been described in patients with a ventricular-peritoneal shunt (Mccallum *et al*, 1988) and one case of metastasis to the tibia has been published (Hayakawa *et al*, 1979). The symptom duration of patients with tumours located in the cerebellopontine angle was longer than in other patients.

**Figure 2:**
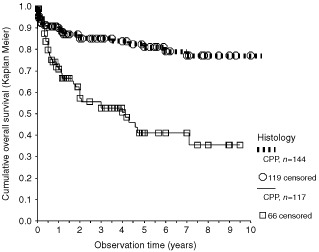
Survival of patients published as CPP with CPC patients. Only patients with observation times >0 and clear histological allocation were included. Symbols indicate end of observation times of surviving patient (=censored data). Statistically significant: *P*<0,0005, log rank test.

When survival was used as endpoint, patients with observation time equaling 0 were excluded. This left 266 patients for analysis. In these patients, the prognostic relevance of the available variables were assessed. In an univariant analysis, histology was a significant prognostic factor (Figure 2

). One, five, and 10 year projected survival rates of plexus carcinoma patients were 71, 41, 35% in comparison to CPP patients with 90, 81, 77%, respectively (Figure 2, *P*<0.0005 log-rank test). Some tumours were primarily described as ‘atypical choroid plexus papilloma’. In second surgeries or autopsies of these patients, a repeat histology was frequently reported as CPC. The survival curve of those ‘atypical CPP’ was between the curves of CPP and CPC, but the number of cases was too small for a meaningful analysis.

**Figure 3:**
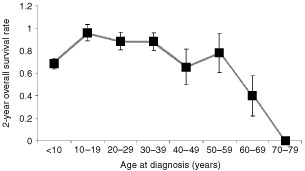
Young children and patients over 59 years of age have a worse prognosis as compared to the age classes in between (*P*<0.0005 log rank test). This included patients, in whom the observation time was >0, and in whom age at diagnosis was documented. Survival rates were calculated as Kaplan–Meier estimates.

When analysing age at diagnosis, the poorest survival was noted for those over 40 years of age, followed by those under 10 years of age, with the best outcome for those aged 10–40 years (Figure 3

). The gender and location parameters did not have prognostic relevance. In the total group of CPT, metastasis was a significant factor in survival time (*P*=0.02), however, metastases were more frequent in CPC, and in subgroup analyses the numbers became too small for meaningful analysis.

**Figure 4:**
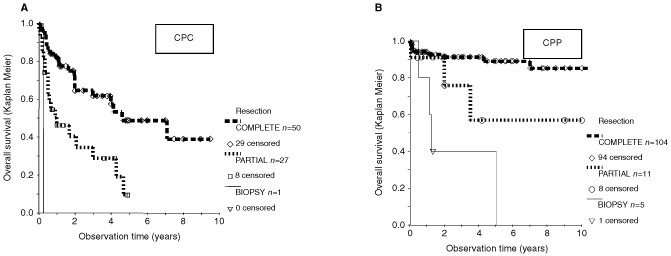
In both, CPC, (A) and CPP (B), the type of surgery was a statistically significant prognostic factor (*P*=0.0001, and *P*<0.00005, respectively, log rank tests).

**Figure 5:**
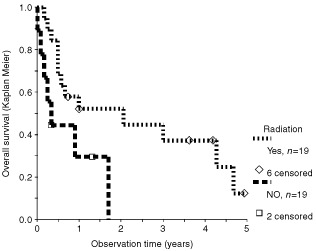
Influence of irradiation on survival with incompletely resected CPC. Inclusion factors for this analysis were: Observation time >0, histology CPC, surgery well documented, less than gross total resection. The difference in favour of irradiation is marginally statistically significant (*P*=0.029, log rank test).

Treatment modalities analysed in this study included surgery, radiotherapy, and chemotherapy. Of those, surgery showed the most striking differences (Figure 4

). CPP patients with complete resection had a 10-year survival rate of 85% (±10% standard deviation). This compared to 56% with less than gross total resection and to a one year survival rate of only 50% in patients after biopsy (*P*=0.002). CPC patients had a 2-year survival rate of 72% (±10%) *vs* 34% (±10%) for those with a gross total resection or an incomplete resection, respectively. For the analysis of irradiation, the variable ‘malignancy’ and ‘surgery’ were used to create subgroups. For CPC patients, those that received irradiation did better than those without irradiation in both subgroups, after complete and incomplete resection (Figure 5

). In the subgroup of patients with CPC who had gross total resection, there were 25 patients who further received irradiation and 24 patients who did not receive irradiation. The comparison of these two groups showed a significant benefit for patients receiving irradiation (Wolff *et al*, 1999). The data of patients with incomplete resection are shown in Figure 5. The survival time of 19 patients receiving irradiation was significantly better (*P*=0.029) than that of nine patients not given irradiation. Response to chemotherapy was well documented in CPC (Table 1

**Table 1:**
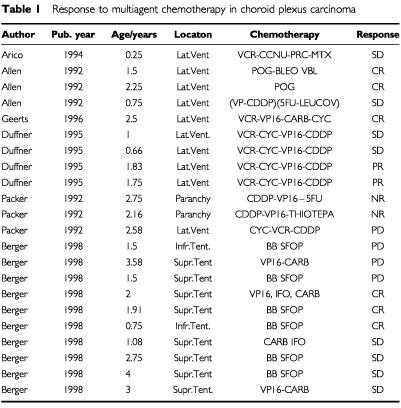
Response to multiagent chemotherapy in choroid plexus carcinoma

). The overall response rate was 8 of 22. With these limited data the impact of chemotherapy on survival could not be sufficiently analysed.

Relapse after treatment was documented in 52 cases. It was not surprising that CPC patients who relapsed after treatment did poorly. The projected 2-year survival rate for these patients was 54%, as compared to 62% for CPC patients in which relapse was not mentioned. By contrast, CPP patients with documented relapse had the same survival as CPP patients without relapse. A further interesting difference between CPP and CPC came up when the year of publication was analysed with respect to prognosis. Surprisingly, there was no improvement of the CPC-prognosis over the years. By contrast the survival of CPP patients published after 1970 was superior to earlier publications (5-year survival rate: 83%, *n*=132 *vs* 35%, *n*=12, significant *P*<0.0005, log-rank test).

## DISCUSSION

This systematic literature review was initiated to determine risk factors and the best management for choroid plexus tumours (CPT). However, a literature review cannot produce the level of evidence that one might wish for since there is no certain way to control publication bias. Whenever possible, clinical treatment decisions should be based on prospective randomised trials, but in the absence of those, a systematic literature review might provide the best possible evidence.

The etiology of some choroid plexus tumours has been linked to SV40 infections (Tabuchi *et al*, 1978a,b; Bergsagel *et al*, 1992; Lednicky *et al*, 1995; Martini *et al*, 1996), but it might also be influenced by other factors such as X-chromosome linked syndromes (Steichen-Gersdorf *et al*, 1993; Sybert *et al*, 1994; Kleihues, 1997). We found significant differences when relating gender to age at diagnosis, tumour location or pathology. Cerebello-pontine angle tumours were associated with older age, benign histology, and female gender. This could suggest a tumour entity linked to a tumour-suppressor gene located on the X-chromosome. Interestingly, the syndromes with defects on the X-chromosome were reported with choroid plexus papilloma (Hamano *et al*, 1991; Steichen-Gersdorf *et al*, 1993; Sybert *et al*, 1994; Trifiletti *et al*, 1995; Zajac *et al*, 1997). By contrast, the infant type of choroid plexus tumours was most frequently located in the lateral ventricles and had a male predominance. Interestingly, the report about SV40 in choroid plexus tumours described children only (Bergsagel *et al*, 1992). Based on this analysis we hypothesised that SV40 is more frequently found in infant tumours and that there is a distinctly different aetiology in adult choroid plexus tumours.

The definitive diagnosis of a choroid plexus-tumour can only be made histologically. The allocation to either CPP or CPC might be debatable in individual cases. In fact, others have found a significant number of tumours changing the allocated diagnosis, when reviewed in a reference centre (Berger *et al*, 1998). Despite this, our data show that the published histological diagnosis is a significant risk factor, even when reference pathology is missing in most cases. Surgery is therefore required for the diagnosis. Tumour resection is also a powerful therapeutical step. In both diagnoses, CPC and CPP, the prognosis was better in completely resected tumours. Especially in the case of CPC patients (Packer *et al*, 1992), the positive relevance of surgery appears to be greater than in most other brain tumours. This supports a second surgery when the post surgical radioimaging shows residual tumour.

In CPC patients adjuvant treatment should follow surgery. Response to irradiation has been demonstrated in at least five patients with residual tumour after surgery (Carrea and Polak, 1977; Naguib *et al*, 1981; Griffin *et al*, 1988; Hashizume *et al*, 1995; Wolff *et al*, 2001a,b). Based on our analysis, patients with gross total resection should indeed receive irradiation. We confirmed significant improved survival for patients with CPC who received irradiation in both subgroups (a) with residual tumour (Figure 5) and (b) after gross total resection (Wolff *et al*, 1999). However, radiation is to be avoided in young children, in whom long term sequelae are severe. This leaves chemotherapy as adjuvant modality for the majority of patients. Chemotherapy can result in shrinkage of CPC tissue (Table 1), and chemotherapy has been recommended by some authors (Duffner *et al*, 1995; Geerts *et al*, 1996), but the experience with this modality has been limited, and evidence for an impact on the number of long term surviving patients is still missing. The numbers are simply too small to provide such evidence. Similarly, the relative contribution of the various single agents to response cannot be evaluated. A clinical study addressing that question will need a patient population larger than any of the current national paediatric oncology groups. This should therefore be done in an intercontinental setting. The fact that prognosis of patients published prior to 1970 is unchanged to the prognosis in the most recent publications warrants the start of such an effort. Recurrence of CPC has to be judged incurable with conventional treatment, and is therefore an indication for experimental treatment.

CPP treatment should be different. Surgery appears to be the most important modality in plexus papilloma (Raimondi and Gutierrez, 1975; Schijman *et al*, 1990; Sharma *et al*, 1994; Tacconi *et al*, 1996; Costa *et al*, 1997). Guidetti and Spallone (1981) reported 50% survivors after surgery only already in 1981. In infants, plexus papillomas are the brain tumours with the best prognosis (Mcgirr *et al*, 1988; Tomita *et al*, 1988; Buetow *et al*, 1990). In contrast to CPC, there is no published evidence yet that adjuvant treatment has any impact on survival of CPP patients. Recurrences may occur as late as 11 years after diagnosis (Buetow *et al*, 1990), which is much later than predicted by the Collins rule (Valladares *et al*, 1980). However, in our analysis, the prognosis of patients with CPP is still good when these patients relapse. The better prognosis documented in publications after 1970 is most likely caused by improvement of surgical and intensive care techniques. Therefore, the data collected in this review further support a ‘wait and see’ policy after first tumour resection.

In conclusion, the data generated in this meta-analysis supports the hypothesis of distinct choroid plexus tumour subtypes which differ in variables such as gender, age at diagnosis, prognosis, location and malignancy. The previously known importance of gross total resection was confirmed by this analysis. In addition, evidence was provided that adjuvant therapy increases survival in CPC patients. This work was initiated by an international consensing discussion, which has resulted in an international study to standardise and optimise the therapy.
